# Self-regulated 1-butanol production in *Escherichia coli* based on the endogenous fermentative control

**DOI:** 10.1186/s13068-016-0680-1

**Published:** 2016-12-19

**Authors:** Rex C. Wen, Claire R. Shen

**Affiliations:** Department of Chemical Engineering, National Tsing Hua University, 101, Section 2, Kuang-Fu Road, Hsinchu, 30013 Taiwan

**Keywords:** 1-Butanol, Redox balance, Fermentation regulation, Metabolic engineering, Synthetic biology

## Abstract

**Background:**

As a natural fermentation product secreted by *Clostridium* species, bio-based 1-butanol has attracted great attention for its potential as alternative fuel and chemical feedstock. Feasibility of microbial 1-butanol production has also been demonstrated in various recombinant hosts.

**Results:**

In this work, we constructed a self-regulated 1-butanol production system in *Escherichia coli* by borrowing its endogenous fermentation regulatory elements (FRE) to automatically drive the 1-butanol biosynthetic genes in response to its natural fermentation need. Four different cassette of 5′ upstream transcription and translation regulatory regions controlling the expression of the major fermentative genes *ldhA*, *frdABCD*, *adhE*, and *ackA* were cloned individually to drive the 1-butanol pathway genes distributed among three plasmids, resulting in 64 combinations that were tested for 1-butanol production efficiency. Fermentation of 1-butanol was triggered by anaerobicity in all cases. In the growth-decoupled production screening, only combinations with formate dehydrogenase (Fdh) overexpressed under FRE_*adhE*_ demonstrated higher titer of 1-butanol anaerobically. In vitro assay revealed that 1-butanol productivity was directly correlated with Fdh activity under such condition. Switching cells to oxygen-limiting condition prior to significant accumulation of biomass appeared to be crucial for the induction of enzyme synthesis and the efficiency of 1-butanol fermentation. With the selection pressure of anaerobic NADH balance, the engineered strain demonstrated stable production of 1-butanol anaerobically without the addition of inducer or antibiotics, reaching a titer of 10 g/L in 24 h and a yield of 0.25 g/g glucose under high-density fermentation.

**Conclusions:**

Here, we successfully engineered a self-regulated 1-butanol fermentation system in *E. coli* based on the natural regulation of fermentation reactions. This work also demonstrated the effectiveness of selection pressure based on redox balance anaerobically. Results obtained from this study may help enhance the industrial relevance of 1-butanol synthesis using *E. coli* and solidifies the possibility of strain improvement by directed evolution.

**Electronic supplementary material:**

The online version of this article (doi:10.1186/s13068-016-0680-1) contains supplementary material, which is available to authorized users.

## Background

Bio-based 1-butanol production from renewable resources has been an important research thrust in recent years for its potential application as transportation fuel and drop-in chemical feedstock [1, 2]. Natural biosynthesis of 1-butanol in *Clostridium* species occurs by acetone–butanol–ethanol (ABE) fermentation via a series of reduction steps of CoA-linked intermediates. While the native producer *Clostridium* remains as the major workhorse for the production of 1-butanol on the industrial scale [3–8], engineering and characterization of the clostridial CoA-dependent pathway in various heterologous hosts have been extensively performed to decipher pathway bottleneck and address its limitation in recombinant systems [9–13]. Production titer and industrial practicality of heterologous 1-butanol synthesis have been significantly improved by many metabolic engineering approaches, such as replacement of inefficient enzymes [13, 14], creation of synthetic driving forces [15, 16], development of co-culturing system [17], utilization of inducer-free promoter [18–20], and analysis of system-level pathway inefficiency [18, 20, 21], reaching the highest productivity of 5–6 g/L/d so far using *Escherichia coli* in bench-scale flasks. In addition to the CoA-dependent pathway, other synthetic pathways based on amino acid biosynthesis [22] and reverse β-oxidation [23] were also explored as the alternative 1-butanol production system.

**Fig. 1:**
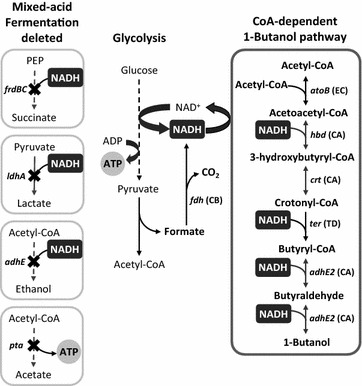
Synthetic 1-butanol pathway expressed in the mixed-acid fermentation mutant. The mixed-acid mutant strain (Δ*ldhA* Δ*frdBC* Δ*adhE* Δ*pta*) relies on the CoA-dependent 1-butanol pathway to recycle the NADH generated in glycolysis and to restore anaerobic growth. Different microbial sources of the 1-butanol pathway enzymes are shown in the parenthesis: EC*, Escherichia coli*; CA, *Clostridium acetobutylicum*; TD, *Treponema denticola*; CB, *Candida boidinii*

In *Clostridium*, 1-butanol is synthesized as a natural fermentation product and along with ethanol and acetone serves as organic electron sink under anaerobic condition. Similarly, the facultative anaerobe *E. coli* responds to a decrease of oxygen by triggering mixed-acid fermentation of succinate, lactate, and ethanol to recycle the excess NADH as aerobic respiration ceases. In this work, we aimed to engineer a self-regulated 1-butanol fermenting strain of *E. coli* by borrowing its native fermentation regulatory system to drive the synthetic 1-butanol production as the sole NADH outlet anaerobically. Aerobically, the reducing power generated in glycolysis and the tricarboxylic acid (TCA) cycle is recycled via oxidative respiration with concomitant production of ATP. Under anaerobic condition, the lack of external electron acceptor results in the stalling of respiration; as a result, the NADH must be oxidized by fermentation reactions in order for glycolysis to proceed. Since ATP production is obligately coupled to the activity of glycolysis in the absence of respiration, regeneration of NAD^+^ by fermentation is crucial for cell survival under anaerobic condition. As shown in the previous studies [15], deletion of the mixed-acid fermentation pathways (Δ*ldhA* Δ*frdBC* Δ*adhE*) led to complete abolishment of *E. coli* growth anaerobically. Growth can be restored by introduction of a NADH outlet such as the synthetic 1-butanol pathway [15] to balance the NADH generation and consumption (Fig. 1). The need to recycle NADH under anaerobic condition has long been utilized as an adaptive evolution system to improve pathway efficiency for various NADH-dependent products such as succinate [24–26], ethanol [27, 28], l-alanine [29], and (D/L)-lactate [30, 31].

**Fig. 2:**
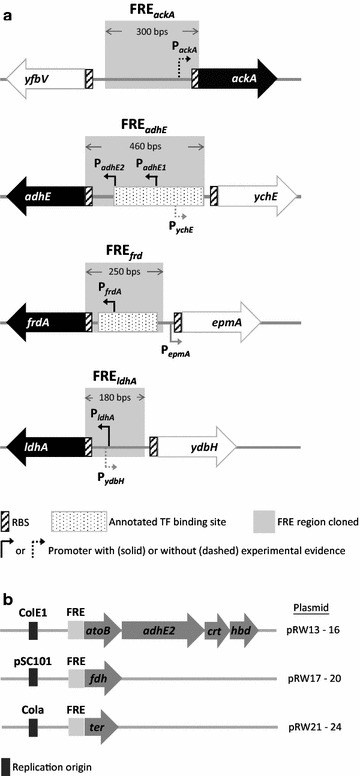
Cloning of the endogenous FRE_*ldhA*_, FRE_*frd*_, FRE_*adhE*_, and FRE_*ackA*_ to drive the genes for 1-butanol synthesis. **a** Coverage of the native FRE cloned (*solid gray block*) upstream of each fermentative gene (*black arrow block*) in *E. coli*, including the endogenous transcription factor-binding site, promoter, and ribosomal-binding site. The neighboring genes are denoted by hollow *gray arrow blocks* while the neighboring promoters are drawn with *gray elbow arrow*. **b** Expression of the 1-butanol pathway genes distributed among three plasmids by the cloned FRE. Operon *atoB*-*adhE2*-*crt*-*hbd* is harbored on plasmid with ColE1 origin, gene *ter* is carried on plasmid with Cola origin, and gene *fdh* is carried on plasmid with pSC101 origin. Each plasmid was paired with the four different FRE individually, creating a total of 12 plasmid constructs (pRW13–pRW24). TF, transcription factor; RBS, ribosomal-binding site

This work aimed to construct a self-regulated 1-butanol production system in *E. coli* based on its natural fermentation need by placing the 1-butanol pathway under the control of* E. coli’s* native regulation. In *E. coli*, lactate, succinate, ethanol, and acetate are the major fermentation products with the terminal reactions catalyzed by lactate dehydrogenase (LdhA), fumarate reductase (FrdABCD), alcohol dehydrogenase (AdhE), and acetate kinase (AckA), respectively. In order to achieve an engineered strain which only ferments 1-butanol, the native fermentation pathways that can serve as electron sink were deleted (Δ*ldhA* Δ*frdBC* Δ*adhE*) to create a NADH driving force for 1-butanol fermentation. The acetate forming reaction was also removed (Δ*pta*) to accumulate acetyl-CoA for driving the reversible thiolase reaction in the condensation direction. These knock-out strategies were shown previously [15] to enhance carbon flux into the 1-butanol pathway (Fig. 1). Four different sets of 5′ upstream transcriptional and translational regulatory elements (hereafter referred to as fermentation regulatory element, or FRE) controlling the expression of *ldhA*, *frdABCD*, *adhE*, and *ackA* were cloned individually to drive the heterologous genes for 1-butanol synthesis. As shown in Fig. 2, the six essential genes for 1-butanol production were expressed from three plasmids (FRE::*atoB*-*adhE2*-*crt*-*hbd*; FRE::*ter*; FRE::*fdh*), and the identical operon structure which demonstrated high production efficiency [15] was used in this study. Previously, the trans-enoyl-CoA-reductase (Ter) from *Treponema denticola* catalyzing the conversion of crotonyl-CoA to butyryl-CoA was hypothesized to be irreversible and the rate-determining enzyme in 1-butanol synthesis [14, 15], while expression of the formate dehydrogenase (Fdh) from *Candida boidinii* was shown to enhance production efficiency by balancing the NADH requirement of the 1-butanol pathway [15, 18, 20]. Thus, it is also desirable to express Ter and Fdh under different FRE to allow separate tuning of their activity and examination of its effect on 1-butanol synthesis. With four different FRE (FRE_*ldhA*_, FRE_*frd*_, FRE_*adhE*_, FRE_*ackA*_) and three plasmids, a total of 12 FRE::gene constructs were cloned (Fig. 2) and 64 combinations (4 × 4 × 4) were tested for 1-butanol production efficiency.

**Fig. 3:**
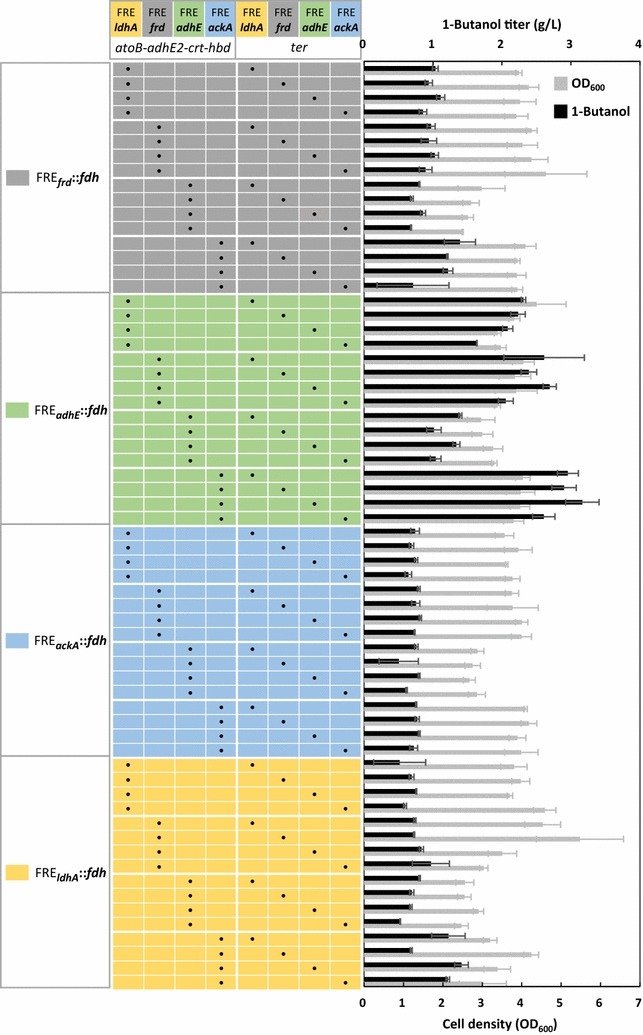
Screening of 1-butanol production driven by the different FRE combinations. Strain Δ*ldhA* Δ*frdBC* Δ*adhE* Δ*pta* was transformed with the three plasmids carrying the operon *atoB*-*adhE2*-*crt*-*hbd*, gene *ter,* and *fdh* expressed by the different FRE. Since each plasmid has four possible FRE to pair with, a total of (4 × 4 × 4) 64 combinations was resulted. Genotype (FRE::gene) of the three plasmids is boxed by *gray borders* on the *top* and on the *left*. Each unique FRE is highlighted by *color blocks*. The *dot* in the table indicates the specific plasmid (FRE::gene) combination harbored in each strain. Production results from the 64 different strains are grouped according to the FRE::*fdh* combination shown on the* left*. Cells were grown in TB medium aerobically to an OD_600_ of about 4 then switched into anaerobic condition to induce 1-butanol fermentation. Samples were taken after 24 h of anaerobic switch

Introduction of the synthetic 1-butanol pathway under the control of native FRE into strain Δ*ldhA* Δ*frdBC* Δ*adhE* Δ*pta* enabled fermentation of 1-butanol upon switch into anaerobic state. Utilization of the multi-copy plasmid system allowed higher expression of pathway genes and quicker assessment of the 1-butanol production efficiency driven by the different FRE combos. Here, we screened the 64 FRE::gene combinations for their potential of anaerobic 1-butanol synthesis under the growth-decoupled (two-phase) production condition. By analyzing their corresponding production pattern, several FRE::gene combinations that had significant impact on 1-butanol synthesis were identified. Compared to *ter* and the *atoB*-*adhE2*-*crt*-*hbd* operon, expression level of *fdh* played a more important role in determining the 1-butanol productivity since only combinations with FRE_*adhE*_::*fdh* could reach a 1-butanol titer above 3 g/L in 24 h anaerobically (Fig. 3). Significantly higher in vitro activity of Fdh was detected from the high-producing strain harboring FRE_*adhE*_::*fdh* compared to its counterparts. Based on the highest 1-butanol producer, we observed similar productivity of 1-butanol under micro-aerobic condition, indicating that gene expression from the native FRE can be sufficiently induced without complete removal of oxygen. Medium analysis revealed that supplementation of rich nutrients is still essential for efficient production of 1-butanol via FRE-controlled expression. In this FRE-based production system, timing of anaerobic switch is crucial since the native fermentation reactions are generally induced by the lack of oxygen. Switching the cells to anaerobic condition at different stage of their growth revealed that exposure to anaerobicity prior to significant accumulation of biomass is necessary to achieve high productivity of 1-butanol. With the plasmids maintained by the selection pressure based on NADH balance, we achieved stable production of 1-butanol anaerobically without the need of an inducer or antibiotics. The best-performing strain (FRE_*ackA*_::*atoB*-*adhE2*-*crt*-*hbd*, FRE_*adhE*_::*ter*, FRE_*adhE*_:: *fdh*) demonstrated a 1-butanol titer of 10 g/L in 24 h and a yield of 0.25 g/g by concentrated cells in bench-scale test tubes.

## Results and discussion

### Cloning of FRE_*ldhA*_, FRE_*frd*_, FRE_*adhE*_, and FRE_*ackA*_ to drive the genes for 1-butanol synthesis

In order to utilize the endogenous control of fermentation reactions to construct a self-regulated 1-butanol production system in *E. coli*, we borrowed the native control elements originally driving the genes for mixed-acid fermentation (lactate, succinate, acetate, and ethanol) and used them to auto-regulate 1-butanol biosynthesis on the transcriptional and translational level. Four unique cassette of 5′ upstream transcriptional and translational regulatory elements (FRE) controlling the expression of *ldhA*, *frdABCD*, *adhE*, and *ackA* were cloned to drive the heterologous genes in the 1-butanol pathway. Based on the ECOCYC annotation [32], the cloning strategy for each of the FRE cassettes was to include the promoter, ribosomal-binding site (RBS), and the entire transcription factor (TF)-binding sites, regardless for activator or repressor, to fully utilize the native transcriptional and translational control of fermentation. We also tried to minimize overlap with neighboring promoter and RBS if possible. Thus, the FRE clones covered the sequence immediately upstream of the start codon for the fermentative genes to the furthest annotated TF-binding site, or as far as possible without intercepting the RBS for the neighboring gene (Fig. 2). At the end, 300 base pairs (bp), 460, 250, and 180 bp of the corresponding upstream region were cloned to represent FRE_*ackA*_, FRE_*adhE*_, FRE_*frd*_, and FRE_*ldhA,*_ respectively. It is interesting to note that FRE_*adhE*_ contained two annotated promoters as shown in ECOCYC. It is also noted that in the case of FRE_*adhE*_ and FRE_*ldhA*_, the cloned coverage inevitably contained the putative promoter for the neighboring gene running in the opposite direction.

The four endogenous FRE were cloned individually to control expression of the enzymes for 1-butanol production. As shown in Figs. 1 and 2, the six essential genes (*atoB*, *hbd*, *crt*, *ter*, *adhE2*, and *fdh*) involved in the 1-butanol pathway were cloned onto three different plasmids, with the operon *atoB*-*adhE2*-*crt*-*hbd* harbored on high copy (ColE1 origin), *ter* on medium copy (Cola origin), and *fdh* (pSC101 origin) on low copy. To explore the effect of different FRE combos on the regulation and efficiency of 1-butanol production, the four endogenous FRE were cloned individually onto each of the three plasmids harboring the 1-butanol biosynthetic genes, resulting in 12 (4 × 3) FRE::gene constructs at the end (Fig. 2 and Table 1). With a total of 12 plasmids cloned, 64 different combinations (4 × 4 × 4) were generated and tested for 1-butanol production efficiency.

**Table 1:** Strains and plasmids used in this study

	Genotype	Reference
**Strain**		
BW25113	*rrnB*_T14_ ∆*lacZ*_WJ16_ *hsd*R514 ∆*araBAD*_AH33_ ∆*rhaBAD*_LD78_	[45]
XL-1 blue	*recA1 endA1 gyrA96 thi*-*1 hsdR17 supE44 relA1 lac* [F’ *proAB lacI* ^*q*^ *Z∆M15 Tn10* (Tet^r^)]	Stratagene
JCL16	BW25113/F’ [*traD36 proAB* ^+^ *lacI* ^q^Z∆M15 (Tet^r^)]	[9]
JCL299	JCL16 ∆*ldhA* ∆*adhE* ∆*frdBC* ∆*pta*	[9, 15]
**Plasmid**		
pRW13	FRE_*ackA*_::*atoB* (EC) *adhE2* (CA) *crt* (CA) *hbd* (CA); ColE1 ori; Amp^r^	This study
pRW14	FRE_*adhE*_::*atoB* (EC) *adhE2* (CA) *crt* (CA) *hbd* (CA); ColE1 ori; Amp^r^	This study
pRW15	FRE_*frd*_::*atoB* (EC) *adhE2* (CA) *crt* (CA) *hbd* (CA); ColE1 ori; Amp^r^	This study
pRW16	FRE_*ldhA*_::*atoB* (EC) *adhE2* (CA) *crt* (CA) *hbd* (CA); ColE1 ori; Amp^r^	This study
pRW17	FRE_*ackA*_::*fdh* (CB); pSC101 ori; Cm^r^	This study
pRW18	FRE_*adhE*_::*fdh* (CB); pSC101 ori; Cm^r^	This study
pRW19	FRE_*frd*_::*fdh* (CB); pSC101 ori; Cm^r^	This study
pRW20	FRE_*ldhA*_::*fdh* (CB); pSC101 ori; Cm^r^	This study
pRW21	FRE_*ackA*_::*ter* (TD); Cola ori; Kan^r^	This study
pRW22	FRE_*adhE*_::*ter* (TD); Cola ori; Kan^r^	This study
pRW23	FRE_*frd*_::*ter* (TD); Cola ori; Kan^r^	This study
pRW24	FRE_*ldhA*_::*ter* (TD); Cola ori; Kan^r^	This study
pEL11	P_L_lacO1:: *atoB* (EC) *adhE2* (CA) *crt* (CA) *hbd* (CA); ColE1 ori; Amp^r^	[15]
pIM8	P_L_lacO1:: *ter* (TD); Cola ori; Kan^r^	[15]
pCS138	P_L_lacO1:: *fdh* (CB); pSC101 ori; Cm^r^	[15]

Abbreviations in the parenthesis indicate source of the genes: EC*, Escherichia coli*; CA, *Clostridium acetobutylicum*; TD, *Treponema denticola*; CB, *Candida boidinii*

### Screening of 1-butanol production and analysis of enzyme expression by the different FRE combinations

We anticipated variation in the level of regulation and expression from the four native FRE, of which the combinatorial effect with different pathway genes may lead to high or lower production of 1-butanol. To characterize and compare 1-butanol fermentation efficiency obtained from the 64 combinations, we transformed strain Δ*ldhA* Δ*frdBC* Δ*adhE* Δ*pta* with the 64 different plasmid combos and performed production experiments anaerobically in the TB glucose medium. Here, the growth-decoupled production process was used, where cells were first grown aerobically to stationary phase (OD_600_ ~ 4) then switched into anaerobic condition to induce 1-butanol fermentation. Accumulation of biomass prior to induction is a common fermentation strategy and thus was chosen as the production condition in the screening process.

Figure 3 shows the anaerobic 1-butanol production profile and cell density of the 64 combinations under the growth-decoupled condition. Fermentation of 1-butanol was triggered by anaerobicity with significant variation in the production titer from the different FRE combinations. Cell density had no strong correlation with 1-butanol productivity in this case since biomass was accumulated prior to switching into fermentative condition. As shown in Fig. 3, expression of *fdh* appeared to be the most determining factor in 1-butanol productivity, followed by the *atoB*-*adhE2*-*crt*-*hbd* operon and then *ter*. Fdh catalyzes the oxidation of formate into carbon dioxide and NADH. Only combinations with the FRE_*adhE*_::*fdh* could reach a titer of 2–3 g/L in 24 h, while most of the other combos accumulated less than 1 g/L of 1-butanol. Within the 12 combinations of FRE_*adhE*_::*fdh* (green shaded table section in Fig. 3), expression of the *atoB*-*adhE2*-*crt*-*hbd* operon under FRE_*ackA*_ led to higher 1-butanol titer regardless of which FRE controls the *ter* expression. On the other hand, expression of the *atoB*-*adhE2*-*crt*-*hbd* operon under FRE_*adhE*_ was detrimental to 1-butanol fermentation. Among the high producers with FRE_*adhE*_::*fdh*, coupling of *ter* expression to FRE_*adhE*_ or FRE_*ldhA*_ resulted in better production of 1-butanol. Compared to *fdh*, variation in the expression level of *ter* or the *atoB*-*adhE2*-*crt*-*hbd* operon by the different FRE was less influential on the 1-butanol productivity. Overall, the best-performing strain which demonstrated the highest 1-butanol titer under the growth-decoupled condition was shown to harbor FRE_*adhE*_::*fdh*, FRE_*ackA*_::*atoB*-*adhE2*-*crt*-*hbd*, and FRE_*adhE*_::*ter*. Variation in the 1-butanol production pattern as observed among the 64 combinations suggests differences in the regulation and level of expression from each FRE and the importance of Fdh activity in 1-butanol production.

**Fig. 4:**
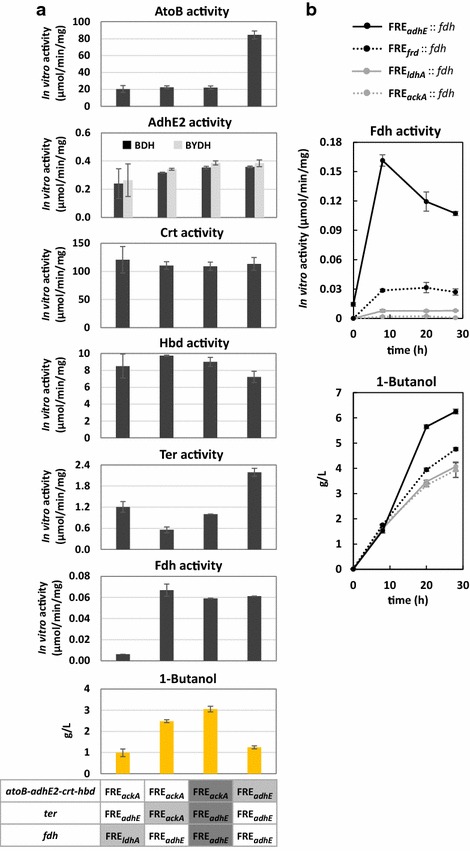
Correlation between 1-butanol productivity and enzyme expression by the different FRE. **a** Comparison of enzyme expressions in the low and high producers of 1-butanol. Two low and two high producers (including the best producer) from the screening experiment were used in this study. The best 1-butanol producer (FRE_*adhE*_::*fdh*, FRE_*ackA*_::*atoB*-*adhE2*-*crt*-*hbd*, and FRE_*adhE*_::*ter*) is shaded with *dark gray* in the table below, while the one particular FRE change in the other three variants is shaded in *light gray*. Strain Δ*ldhA* Δ*frdBC* Δ*adhE* Δ*pta* was used as the host. Identical cultivation procedure for 1-butanol production as in the screening process was followed. Samples were taken and cells were harvested for in vitro assay after 24 h of anaerobic switch. BDH, AdhE2 activity toward butyraldehyde; BYDH, AdhE2 activity toward butyryl-CoA. **b** Time course of Fdh expression regulated by each FRE. The highest 1-butanol producer (FRE_*ackA*_::*atoB*-*adhE2*-*crt*-*hbd*, FRE_*adhE*_::*ter*, FRE_*adhE*_::*fdh*) along with its three other FRE::*fdh* variants were used in this study. All strains contained FRE_*ackA*_::*atoB*-*adhE2*-*crt*-*hbd* and FRE_*adhE*_::*ter* but different FRE::*fdh* as shown in the figure legend. Strain Δ*ldhA* Δ*frdBC* Δ*adhE* Δ*pta* was used as the host. Identical cultivation procedure for 1-butanol production as in the screening process was followed except that cells were switched to anaerobic condition around an OD_600_ of 0.4. Samples were taken at various time points and cells were harvested for in vitro assay of Fdh activity. “Time” indicates time since anaerobic switch

To investigate if the better production performance of 1-butanol from certain FRE combination was a result of enhanced balance between gene expressions or due to upregulation of the entire 1-butanol pathway, we assayed in vitro activity of each overexpressed enzyme using crude extracts prepared from two high producers and two low producers of 1-butanol. Specifically, the best 1-butanol producer was selected as the comparison basis along with three other variants consisting of identical combination but one FRE change for the *atoB*-*adhE2*-*crt*-*hbd* operon, *ter* or *fdh* expression (Fig. 4a). As shown by the assay results, higher production of 1-butanol by certain FRE combinations was not a consequence of elevated expression of the entire 1-butanol pathway. In vitro activity of AdhE2, Crt, Hbd, and Ter was similar between the high and the low producers and appeared to be less influential on the 1-butanol production efficiency. On the contrary, significantly higher AtoB activity or lower Fdh activity was observed from the two low producers individually. It has been well accepted that sufficient expression of Fdh is essential to achieve NADH balance with the 1-butanol pathway [15, 18, 20], which corresponds well to our observation. However, intriguingly, our results show that excessive expression of AtoB was detrimental to 1-butanol synthesis and would lead to lower production titer even under sufficient expression of Fdh (Fig. 4a). The underlying cause remains to be clarified. Overall, these results indicate that the increase in 1-butanol productivity by certain FRE combination was due to a better balance between the gene expressions and not upregulation of the entire pathway.

### Effect of FRE strength on the Fdh expression and 1-butanol production

Because the production level of 1-butanol in the screening experiment showed strong dependency on the type of FRE::*fdh* expression cassette, we set out to investigate how the different FRE regulates gene expression in a time course using Fdh activity as an output. In vitro activity of Fdh from the highest producing combination (FRE_*ackA*_::*atoB*-*adhE2*-*crt*-*hbd*, FRE_*adhE*_::*ter*, FRE_*adhE*_::*fdh*) was assayed and compared to the Fdh activity from the other three strains harboring the identical expression cassettes but with different FRE controlling the *fdh* (FRE_*ldhA*_, FRE_*frd*_, or FRE_*ackA*_). Cells were harvested at various time points upon anaerobic switch and crude extracts were prepared from these four strains during the course of 1-butanol fermentation.

As shown in Fig. 4b, expression of Fdh by the various FRE was promptly induced by anaerobicity and reached the maximum after 8 h of anaerobic switch. Significantly higher activity of Fdh was detected from the best-performing strain harboring FRE_*adhE*_::*fdh*, which slowly dropped as fermentation continued. Compared to FRE_*adhE*_, lower Fdh activity was detected from FRE_*frd*_ followed by FRE_*ldhA*_ throughout the time course, while minimal activity of Fdh was detected in the case of FRE_*ackA*_. This observation shows direct correlation with 1-butanol production efficiency, where higher titer of 1-butanol was achieved from the strains with FRE_*adhE*_::*fdh* and proportionally lower titer of 1-butanol was observed from FRE_*frd*_::*fdh*, FRE_*ldhA*_::*fdh*, and FRE_*ackA*_::*fdh,* in the same order as the level of Fdh activity detected. It also demonstrates that efficient 1-butanol synthesis requires sufficient expression of Fdh to generate enough NADH driving force for channeling carbon flux into the 1-butanol pathway. This result strengthens the conclusion made by Lim et al. [18], where increasing Fdh expression helped balancing the intracellular redox state and improved 1-butanol productivity from glucose. As reflected by the different levels of Fdh activity, FRE_*adhE*_ appeared to exhibit the greatest expression strength throughout the time course followed by FRE_*frd*_, FRE_*ldhA*_, and FRE_*ackA*_. Similar pattern of FRE strength was also observed from the AtoB and Ter activity comparisons in the expression characterization experiment described previously (Fig. 4a). It is worth nothing that the FRE_*adhE*_ region which we cloned consists of two annotated promoter, which may contribute to the higher expression level observed compared to the other three FRE. Cross-checking the expression pattern with the 1-butanol production level from the screening experiment, these results suggest that high expression of Fdh coupled to relatively lower expression of AtoB may be favorable toward 1-butanol synthesis under the growth-decoupled condition.

### Complete removal of oxygen is not required for the induction of 1-butanol fermentation

One of the advantages of placing the 1-butanol pathway genes under the control of native FRE is to grant cells the capability of self-regulating the synthesis of 1-butanol based on its intracellular redox state. To analyze if strict anaerobic condition is necessary to induce expression from the FRE and if the 1-butanol production efficiency is directly proportional to the state of anaerobicity, we performed the production experiment under different levels of aeration. The strain (FRE_*ackA*_::*atoB*-*adhE2*-*crt*-*hbd*, FRE_*adhE*_::*ter*, FRE_*adhE*_::*fdh*) which demonstrated the highest 1-butanol productivity in the anaerobic screening process was used in this case. Various cultivation vessel and culture volume were used in combination with gas exchange to simulate the aerobic, micro-aerobic (tube), micro-aerobic (flask), and anaerobic condition (see “Methods” section for detailed description). Cells were grown to exponential phase aerobically prior to switching into the corresponding cultivation vessel to generate different aeration effects.

**Fig. 5:**
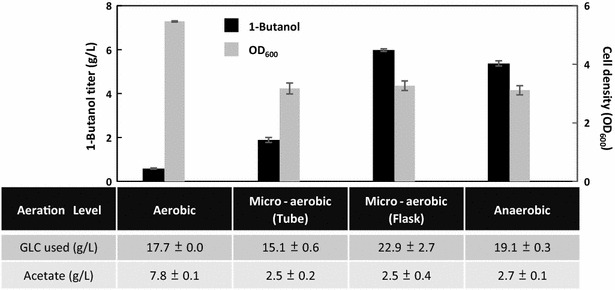
Effect of oxygen level on the FRE-controlled 1-butanol fermentation. Strain Δ*ldhA* Δ*frdBC* Δ*adhE* Δ*pta* transformed with the best combination (FRE_*ackA*_::*atoB*-*adhE2*-*crt*-*hbd*, FRE_*adhE*_::*ter*, FRE_*adhE*_::*fdh*) was used in this study. Cells were grown in TB medium aerobically to mid-log phase then switched into various cultivation vessels to simulate the different oxygen levels as described in “Methods” section. Samples were taken after 24 h of anaerobic switch. GLC, glucose

As shown in Fig. 5, significant increase of oxygen level in the production culture had detrimental effect on 1-butanol synthesis, dropping the production titer from 5.4 g/L in 24 h to 0.6 g/L under aerobic condition. Intermediate level of 1-butanol was achieved in the micro-aerobic (tube) condition, reaching about 2 g/L in 24 h. It is interesting to note that similar productivity and yield of 1-butanol as demonstrated in the strict anaerobic case can also be obtained using the micro-aerobic (flask) condition, indicating that gene expression from the native FRE can be sufficiently induced without complete removal of oxygen. Since the *C. acetobutylicum* AdhE2 used in this study is known to be oxygen labile [33], this result also suggests that the simulated micro-aerobic (flask) condition comprised limited amount of oxygen. Cell density only lowered slightly with decreasing aerobicity and reached similar level (OD_600_ ~ 3) among the oxygen-limiting cases, demonstrating effective growth restoration of the mutant strain Δ*ldhA* Δ*frdBC* Δ*adhE* Δ*pta* by 1-butanol production under those condition. Combined with the observations above, these results demonstrate that efficient induction of 1-butanol synthesis under the FRE system does not require complete elimination of oxygen, which allows for flexibility in the fermentation process design.

### 1-Butanol productivity decreased with removal of complex medium components

Media composition is one of the important aspects during the optimization for an efficient fermentation process. In this study, we demonstrated decent 1-butanol production and anaerobic growth restoration with FRE-based expression in the rich TB medium. To assess if similar behavior of 1-butanol synthesis can be achieved without supplementation of complex nutrients, we repeated the production experiment using the highest producer (FRE_*ackA*_::*atoB*-*adhE2*-*crt*-*hbd*, FRE_*adhE*_::*ter*, FRE_*adhE*_::*fdh*) in minimal medium. M9 medium with or without 5 g/L of yeast extract was tested in this case. Cells were grown aerobically to mid-log phase in the designated medium prior to switching into complete anaerobic condition.

**Fig. 6:**
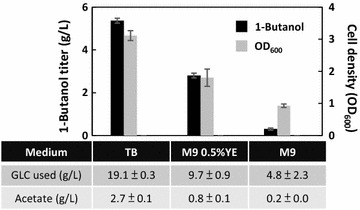
Supplementation of complex nutrient is required to achieve efficient 1-butanol production. Strain Δ*ldhA* Δ*frdBC* Δ*adhE* Δ*pta* transformed with the best combination (FRE_*ackA*_::*atoB*-*adhE2*-*crt*-*hbd*, FRE_*adhE*_::*ter*, FRE_*adhE*_::*fdh*) was used in this study. Samples were taken after 24 h of anaerobic switch. M9 medium with and without 5 g/L of yeast extract (YE) was tested here for 1-butanol production in addition to the Terrific Broth (TB). GLC, glucose

As shown in Fig. 6, the overall titer of 1-butanol synthesis dropped significantly by the removal of complex nutrients from the medium. 1-Butanol production reduced about 50% in the M9Y (M9 with yeast extract) medium and nearly 95% in the M9 medium, reaching 2.8 and 0.3 g/L in 24 h, respectively. However, yield of 1-butanol remained similar in the case of TB and M9Y medium. Interestingly, decent anaerobic cell growth from exponential phase was observed in both the M9Y and M9 medium. Addition of 5 g/L of yeast extract consistently demonstrated beneficial effect for 1-butanol production in minimal media. These results suggest that the presence of complex nutrients such as yeast extract and/or tryptone in the production medium may be essential to achieve efficient 1-butanol synthesis anaerobically regardless of the expression system. This phenomenon has been observed in the anaerobic and micro-aerobic synthesis of 1-butanol previously [9, 14, 15, 21], where removal of complex protein hydrolysate from the culture medium was detrimental toward the production titer [9, 15]. In contrast to other microbial fermentation products such as ethanol, succinate, and lactate [25, 28, 31], recombinant production of 1-butanol remained extremely low in pure minimal medium, despite various engineering efforts. While the underlying reason remains unclear, this could be attributed to the metabolic burden imposed on cells by 1-butanol synthesis as a result of intracellular redox imbalance [18, 21]. High demand of acetyl-CoA and NADH by the 1-butanol pathway (Fig. 1) may also leave cells limited resource for protein synthesis. In this study, the use of native FRE was unable to significantly lower the metabolic demand caused by overexpression of the 1-butanol pathway; nevertheless, the FRE-based expression enabled higher 1-butanol productivity and better growth restoration of the mutant Δ*ldhA* Δ*frdBC* Δ*adhE* Δ*pta* anaerobically in M9Y and M9 medium compared to the P_L_lacO1-based system [9, 15] (see Additional file 1). One of the advantages of anaerobic 1-butanol production using the CoA-dependent pathway in the mixed-acid mutant strain is the potential for selection [15] and serial enrichment of high producers based on its anaerobic growth. Therefore, further fine-tuning of pathway expression and optimization of cellular metabolism in the minimal medium may be attempted via directed evolution.

### Efficiency of 1-butanol production affected by the timing of anaerobic switch

**Fig. 7:**
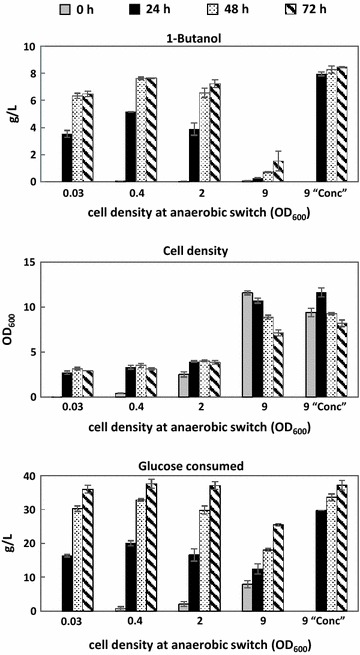
1-Butanol production efficiency affected by the timing of anaerobic switch. Strain Δ*ldhA* Δ*frdBC* Δ*adhE* Δ*pta* transformed with the best combination (FRE_*ackA*_::*atoB*-*adhE2*-*crt*-*hbd*, FRE_*adhE*_::*ter*, FRE_*adhE*_::*fdh*) was used in this study. Cells were grown in TB medium aerobically then switch to anaerobic condition at various cell densities as indicated on the* x*-axis. The label “Conc” describes the condition where cells were first grown anaerobically overnight in TB to induce enzyme expression and then concentrated to an OD_600_ around 9 for subsequent 1-butanol production under anaerobic condition. Adjustment of culture pH and feeding of glucose was performed every 24 h. The figure legend (h) indicates time since anaerobic switch

Accumulation of biomass is a common practice generally performed under aerobic condition to boost the production titer in a fermentation process. In our case, induction of the essential genes for 1-butanol fermentation occurs automatically by the lack of oxygen; therefore, the timing of anaerobic switch becomes an important factor controlling gene expression and thus production efficiency. To examine the performance of 1-butanol fermentation induced at different cell densities, we switched the cells to anaerobic condition at various phases in their life cycle and compared the resulting 1-butanol production titer. The best producer (FRE_*ackA*_::*atoB*-*adhE2*-*crt*-*hbd*, FRE_*adhE*_::*ter*, FRE_*adhE*_::*fdh*) which demonstrated high productivity under the growth-decoupled condition was used in this study. Cells were grown aerobically and then switched to anaerobic condition to induce 1-butanol fermentation at lag phase (OD_600_ ~ 0.03), exponential phase (OD_600_ ~ 0.4), early stationary phase (OD_600_ ~ 2), or late stationary phase (OD_600_ ~ 9). Production of 1-butanol from each test was examined over the course of three days with adjustment of culture pH to neutral and feeding of glucose every 24 h. As shown in Fig. 7, induction of fermentation between lag phase and early stationary phase (OD_600_ of 0.03–2) led to similar production titer of 1-butanol, which plateaued around 6–8 g/L in 72 h. In contrast, switching cells to anaerobic condition at late stationary phase resulted in significantly slower and reduced 1-butanol production, suggesting sluggish protein expression at high cell density.

To analyze if the significant drop of 1-butanol synthesis upon switching at late stationary phase is due to adverse expression of the 1-butanol pathway enzymes, we repeated the production experiment at high cell density with a modified strategy. Cells were first grown overnight anaerobically to induce enzyme expression then concentrated to similar cell density (OD_600_ ~ 9) for anaerobic 1-butanol production. As shown in Fig. 7, synthesis of 1-butanol at high cell density was restored when cells were first grown anaerobically overnight to induce expression of the 1-butanol pathway, reaching around 8 g/L of 1-butanol in 24 h. This result indicates that exposure to oxygen limitation prior to significant accumulation of biomass is essential toward expression of pathway enzymes and 1-butanol productivity. Similar 1-butanol yields were observed from the ones switched at low cell density. It is noted that the plateau of 1-butanol synthesis is often accompanied by the cessation or drop of cell growth. Attempts to further prolong the production by feeding glucose and pH adjustment were not successful, which could be due to the inhibitory effect of 1-butanol toxicity [34, 35].

### Production of 1-butanol in the absence of inducer and antibiotics

Since the mixed-acid mutant strain (Δ*ldhA* Δ*frdBC* Δ*adhE* Δ*pta*) relies on the synthetic 1-butanol pathway to recycle the NADH generated in glycolysis, the plasmids carrying the essential genes for 1-butanol synthesis should be maintained by the selection pressure of NADH balance. To verify if similar production profile of 1-butanol can be achieved without antibiotics, fermentation experiments which demonstrated high productivity were repeated with identical procedure in the absence of antibiotics. In this case, two anaerobic conditions were chosen: (1) production upon anaerobic switch at exponential phase and (2) high-density fermentation using concentrated cell.

**Fig. 8:**
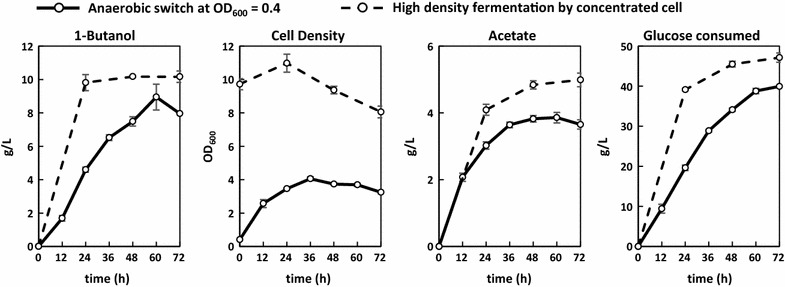
Time course of 1-butanol production in the absence of inducer and antibiotics. Strain Δ*ldhA* Δ*frdBC* Δ*adhE* Δ*pta* transformed with the best combination (FRE_*ackA*_::*atoB*-*adhE2*-*crt*-*hbd*, FRE_*adhE*_::*ter*, FRE_*adhE*_::*fdh*) was used in this study. To examine if similar production efficiency could be maintained in the absence of antibiotics, two of the better performing 1-butanol production conditions were tested here: (1) anaerobic switch at OD_600_ of 0.4 and (2) high-density fermentation by concentrated cell as indicated in the figure legend. Identical cultivation and production procedure to the ones described in Fig. 7 were used. Samples were taken every 12 h (anaerobic switch at exponential phase) or 24 h (high-density culture) anaerobically with adjustment of culture pH and feeding of glucose. “Time” indicates time since anaerobic switch

As shown in Fig. 8, anaerobic production of 1-butanol using the high producer (FRE_*ackA*_::*atoB*-*adhE2*-*crt*-*hbd*, FRE_*adhE*_::*ter*, FRE_*adhE*_:: *fdh*) reached around 10 g/L in 24 h without the need of inducer and antibiotics. Withdrawal of antibiotics did not cause much variation in the production pattern of 1-butanol and anaerobic cell growth (compared to Fig. 7), suggesting decent maintenance of the multi-copy plasmids in the absence of antibiotics anaerobically. Similar to our previous observation, higher 1-butanol productivity was achieved by high-density fermentation using concentrated cell, reaching a peak of 0.4 g/L/h in the first 24 h compared to the 0.2 g/L/h obtained from the production with anaerobic switch at exponential phase. Yield of 1-butanol, on the other hand, remained similar around 0.25 g/g glucose under both cases. Formation of by-product acetate was significant during the production of 1-butanol, approaching 4–5 g/L by the end of fermentation. Under both conditions, 1-butanol synthesis plateaued around the tolerance threshold of 8–10 g/L [36–39] despite intermittent adjustment of culture pH and supply of glucose and yeast extract. Consumption of glucose decreased dramatically along with cell density as 1-butanol synthesis ceased. Anaerobic production of 1-butanol could be restored if the cells were re-inoculated into fresh medium or if the cells were spun down and resuspended with fresh medium, however, reaching only about 40–70% of the original titer (data not shown). This observation suggests that accumulation of inhibitory compounds such as 1-butanol and/or other metabolic wastes in the culture medium might have disrupted membrane function and hindered nutrient uptake as fermentation continued [34, 35]. The toxicity-induced inhibition of cellular metabolism still appears to be the major factor limiting the duration and efficiency of 1-butanol production.

Overall, we demonstrated the efficiency of this inducer-free, antibiotic-free, self-regulated 1-butanol production system based on the native FRE. In the existing studies performed in *E. coli* without gas stripping, various levels of 1-butanol ranging from 0.005 to 6 g/L were achieved in 24 h from the industrially relevant substrates such as glucose [9, 14, 17–20, 40–42], glycerol [9, 43], and galactose [21] under strict anaerobic or micro-aerobic condition. In the high-producing cases, it is consistently observed that efficient synthesis of 1-butanol (~5 g/L in 24 h) under strict anaerobic condition required intensively rich medium such as TB [15, 18], whereas aerobic and micro-aerobic condition allowed comparable 1-butanol productivity using M9Y medium upon strain and pathway engineering [20, 44]. In this study, we achieved similarly high titer (5–6 g/L) of 1-butanol in 24 h via the FRE-based expression anaerobically. In addition, productivity of 1-butanol can be further elevated by high-density fermentation using concentrated cell, reaching 10 g/L in 24 h. This is by far the highest recombinant 1-butanol titer reported in 24 h, even compared to other systems using concentrated cell (~1 g/L in 24 h) [40, 42] or performed with pH-controlled bioreactor coupled to gas stripping (4–6 g/L in 24 h) [15, 44]. Our results also demonstrate the effectiveness of FRE-based expression and its potential application to other bio-based chemical production.

## Conclusions

In this study, we successfully engineered a self-regulated 1-butanol production system by mimicking the natural fermentation response in *E. coli*. The four native FRE originally controlling enzyme expression for the major fermentation reactions were cloned to drive expression of the heterologous 1-butanol pathway. In contrast to individual examination of expression strength from the four different FRE, we screened all the 64 possible FRE::gene combinations anaerobically and identified the group of high producers of 1-butanol. Inspection of the production and expression pattern resulted from the combinatorial effect of the different FRE revealed that 1-butanol productivity is strongly influenced by Fdh and AtoB expression relative to the other pathway enzymes. Sufficient activity of Fdh for additional NADH generation is essential toward anaerobic 1-butanol synthesis. The best-performing combination was identified to be FRE_*adhE*_::*fdh*, FRE_*ackA*_::*atoB*-*adhE2*-*crt*-*hbd*, and FRE_*adhE*_::*ter*.

Characterization of the FRE-based expression indicated that induction of pathway enzymes can be sufficiently achieved without complete removal of oxygen, which adds flexibility to the system. Nevertheless, switching cells to oxygen-limiting condition prior to significant accumulation of biomass appeared to be crucial for enzyme synthesis by the native FRE and 1-butanol productivity. The engineered strain demonstrated a 1-butanol productivity of 0.4 g/L/h and 60% of maximum theoretical yield in the high-density fermentation anaerobically without the addition of an inducer or antibiotic. Although supplementation of complex nutrients is still required for efficient production, the use of native FRE for expression of the synthetic 1-butanol pathway improved anaerobic growth restoration of the mixed-acid mutant close to that of the wild-type strain in TB and M9Y medium. This observation suggests that the FRE-based expression mildly alleviated the metabolic stress commonly associated with strong constitutive and inducible promoter systems. With the suboptimal anaerobic growth observed in minimal medium, further fine-tuning of pathway expression and/or resource distribution toward 1-butanol synthesis in minimal medium may be achieved by directed evolution.

## Methods

### Chemicals and reagents

All chemicals and reagents were purchased from Thermo Scientific (Pittsburgh, PA) or Sigma-Aldrich (Saint Louis, MO) unless otherwise specified. Taq DNA ligase, Phusion High-Fidelity DNA polymerase, and T5 exonuclease were obtained from New England Biolabs (Ipswich, MA). Oligonucleotides were purchased from IDT (San Diego, CA). KOD DNA polymerase was purchased from EMD Chemicals (San Diego, CA).

### Bacterial strains

*Escherichia coli* BW25113 (*rrnB*_*T14*_ ∆*lacZ*_*WJ16*_ *hsdR514 *∆*araBAD*_*AH33*_ ∆*rhaBAD*_*LD78*_) was designated as the wild-type (WT) [45]. XL-1 Blue (Stratagene, La Jolla, CA) was used to propagate all plasmids. Construction of the strain JCL16 (BW25113 with *lacI* ^q^ provided on F’) and JCL299 was described previously [9].

### Plasmid construction

All plasmids were created by the Gibson isothermal DNA assembly method [46] using purified PCR fragments. A list of plasmids is shown in Table 1 and the primers used are shown in Additional File 2. The four individual FRE upstream of the fermentative genes *ldhA*, *adhE*, *frdABCD*, and *ackA* were amplified from the genomic DNA of *E. coli* BW25113 using primers BuOH 1–8 (for pRW 13–16), BuOH 13–20 (for pRW 17–20), and BuOH 23–30 (for pRW 21–24).

To create pRW13–pRW16, the vector backbone (ColE1 ori, Amp^r^) and the operon *atoB*-*adhE2*-*crt*-*hbd* were amplified separately with primers BuOH 9–12 using plasmid pEL11 [15] as the template. The resulting PCR products were gel-purified and independently assembled with the four different FRE fragments. To create pRW17–pRW20, the vector backbone (pSC101 ori, Cm^r^) and the gene *fdh* were amplified together with primers BuOH 21 and 22 using plasmid pCS138 [15] as the template. The resulting PCR products were gel-purified and independently assembled with the four different FRE fragments. To create pRW21–pRW24, the vector backbone (Cola ori, Kan^r^) and the gene *ter* were amplified together with primers BuOH 31 and 32 using plasmid pIM8 [15] as the template. The resulting PCR products were gel-purified and independently assembled with the four different FRE fragments.

### Production media

Unless otherwise specified, production of 1-butanol was performed in terrific broth (TB) (12 g tryptone, 24 g yeast extract, 2.31 g KH_2_PO_4_, 12.54 g K_2_HPO_4_, 4 ml glycerol per liter of water) supplemented with 30 g/L of glucose. In the case of medium analysis, M9 medium (12.8 g Na_2_HPO_4_·7H_2_O, 3 g KH_2_PO_4_, 0.5 g NaCl, 1 g NH_4_Cl, 1 mM MgSO_4_, 1 mg vitamin B1 and 0.1 mM CaCl_2_ per liter of water) without or with 5 g/L of yeast extract (M9Y) was used. Addition of 1000X Trace Metal Mix A5 (2.86 g H_3_BO_3_, 1.81 g MnCl_2_·4H_2_O, 0.222 g ZnSO_4_·7H_2_O, 0.39 g Na_2_MoO_4_·2H_2_O, 0.079 g CuSO_4_·5H_2_O, 0.049 g Co(NO_3_)_2_·6H_2_O per liter water) was performed in both M9 and M9Y medium. No inducer was used in all cases.

### Cultivation condition for 1-butanol production

For the 1-butanol production experiments, single colonies were picked from LB plates and inoculated into 2 mL of LB medium contained in test tubes with the appropriate antibiotics (ampicillin 100 μg/mL, kanamycin 50 μg/mL, and chloramphenicol 50 μg/mL). The overnight culture grown in LB at 37 °C in a rotary shaker (250 rpm) was then inoculated (1% v/v) into fresh TB containing 30 g/L of glucose and appropriate antibiotics unless otherwise specified. The cultures were then grown aerobically at 37 °C and switched to anaerobic condition to induce 1-butanol fermentation at various cell densities as indicated. Anaerobic condition was used for 1-butanol production in all cases except for the aeration analysis. To generate the anaerobic state, 2 mL of culture was transferred to 10 mL BD (BD Biosciences, San Jose, CA) Vacutainer sealed tube. Oxygen was evacuated from the headspace inside the anaerobic transfer chamber with nitrogen and hydrogen using procedure described in [15]. Samples were taken inside the anaerobic chamber if long-term production was performed.

The following describes the detailed cultivation procedure for each of the experiments. For the anaerobic screening of 1-butanol production, cells were switched to anaerobic condition at OD_600_ of around 3–4. To characterize the aeration effect, cells were first grown aerobically in the 250 mL baffled flask and then switched to various cultivation vessels at an OD_600_ of 0.4 as follows: 25 mL of culture transferred to 250 mL screwed cap flask (aerobic), 2 mL of culture transferred to 10 mL Vacutainer sealed tube WITH evacuation of the headspace (anaerobic), 2 mL of culture transferred to 10 mL Vacutainer sealed tube WITHOUT evacuation of the headspace (micro-aerobic “tube”), and 150 mL of culture transferred to 250 mL screwed cap flask (micro-aerobic “flask”). Screwed or sealed caps had to be used to prevent extensive loss of 1-butanol due to its evaporative nature under rigorous shaking. Therefore, except for the case of “anaerobic,” the amount of “headspace” inside the cultivation vessel was used as a rough estimate of aerobicity. For medium analysis, 1% (v/v) of the overnight cell was inoculated into TB, M9Y, or M9 medium and grown aerobically to an OD_600_ of 0.4 prior to switching into anaerobic condition. In the anaerobic growth rescue experiment, 1% (v/v) of the overnight cell was inoculated into M9Y or M9 medium and switched into anaerobic condition immediately. In all cases with M9 medium, cells were first grown in M9 medium overnight prior to fresh inoculation again for anaerobic growth rescue or production.

To test the timing of anaerobic switch, the inoculated culture was either immediately switched to anaerobic condition at an OD_600_ of 0.03 or grown aerobically in the 250 mL baffled flask and then switched to anaerobic condition at an OD_600_ of 0.4, 2, or 9. In the case of “concentrated,” cells were first grown anaerobically overnight in TB at a starting OD_600_ of 0.4. The resulting anaerobic culture was then centrifuged and resuspended with fresh TB while concentrating the cell density to an OD_600_ of around 9. An initial glucose concentration of 60 g/L was used for all of the experiments with concentrated cells. Linear feeding of 10 g/L of glucose and adjustment of culture pH to 7 using 10 M NaOH was performed anaerobically every 24 h.

For 1-butanol production without antibiotics, identical procedure as described above was used except no antibiotic was added. It is noted that the antibiotics were used in the overnight culture. Intermittent feeding of glucose was performed anaerobically every 24 h to maintain glucose level above 20 g/L. Adjustment of culture pH to 7 was performed every 24 h.

### Quantification of metabolites

Samples were centrifuged or filtered to gather the supernatant for GC and HPLC analysis. The amount of 1-butanol produced was quantified by gas chromatograph (GC) equipped with a flame ionization detector (FID). The system is a Shimadzu GC-2010 plus with an AOC-20i auto-injector and an AOC-20s auto-sampler. The separation of alcohol compounds was performed by TG-WaxMS GC column (30 m, 0.32 mm i.d., 0.50 μm film thickness) purchased from Thermo Scientific. GC oven temperature was initially held at 60 °C for 2 min and raised with a gradient of 10 °C/min until 85 °C and held for 2 min. And then it was raised with a gradient of 45 °C/min until 230 °C and held for 1 min. Helium was used as the carrier gas. The injector was maintained at 225 °C and the detector was maintained at 235 °C. The supernatant (1 μL) of culture broth was injected in split injection mode (1:15 split ratio) using 2-methyl-1-pentanol as the internal standard.

To measure concentration of glucose and organic acids, filtered supernatant was applied to an Agilent 1260 HPLC equipped with an auto-sampler and a BioRad (Biorad Laboratories, Hercules, CA) Aminex HPX87H column (5 mM H_2_SO_4_, 0.6 mL/min, column temperature at 50 °C). Glucose was measured with refractive index detector, while organic acids were detected using a photodiode array detector at 210 nm.

### In vitro assay using crude cell extract

All spectrophotometric assays were performed using the Biotek Eon microplate reader at 30 °C under aerobic condition. The reaction mixture volume was 0.2 mL. Protein concentrations were determined by the Bradford assay (Biorad) using BSA as standards. All reactions were initiated by the addition of the crude cell extract.

For the comparison of enzyme expressions in the low and high producers (Fig. 4a), the best 1-butanol producing strain (FRE_*adhE*_::*fdh*, FRE_*ackA*_::*atoB*-*adhE2*-*crt*-*hbd*, and FRE_*adhE*_::*ter*) was selected along with three other variants consisting of identical combination but one FRE change for the *atoB*-*adhE2*-*crt*-*hbd* operon, *ter* or *fdh* expression. Cells carrying the corresponding plasmids were cultured in TB according to the same procedure as described under 1-butanol production and switched to anaerobic condition at OD_600_ of 3–4 (same as the screening experiment). Cells were harvested after 24 h by centrifugation at 13,000 rpm for 10 min and the resulting pellet was resuspended in 0.5 mL of 0.1 M potassium phosphate buffer (pH 7.4). The resuspended culture was then mixed with 0.5 mL of glass beads (Biospec) and homogenized using a mini bead beater (Biospec) aerobically. Part of the homogenated lysate was saved as the total crude extract (for the AdhE2 assay), including the soluble and insoluble fraction. The rest of lysate was centrifuged at 13,000 rpm for 10 min at 4 °C and the supernatant containing the soluble protein was collected. The in vitro assay of AtoB, AdhE2, Crt, Hbd, and Ter was performed and the activity was calculated using identical method and reaction recipe as described previously [15], except that the AdhE2 assay was conducted using total crude extract instead of the soluble fraction. The Fdh assay was performed using the same reaction mixture and method as described below.

For the time course of Fdh activity, the four strains carrying the identical expression cassette for operon *atoB*-*adhE2*-*crt*-*hbd* (pRW13) and *ter* (pRW22), but different FRE for *fdh* expression (pRW 17–20) were cultured in TB according to the same procedure as described under 1-butanol production and switched to anaerobic condition at OD_600_ of 0.4. At various time points since anaerobic switch (0, 8, 20, and 28 h), cells were harvested by centrifugation at 13,000 rpm for 10 min and the resulting pellet was resuspended in 0.5 mL of 0.1 M potassium phosphate buffer (pH 7.4). The crude extract containing the soluble fraction was then prepared using the identical procedure as outlined above. The Fdh activity was measured by monitoring the increase of absorption at 340 nm, corresponding to the generation of NADH. The reaction mixture contained 200 mM sodium formate and 10 mM NAD^+^ in 0.1 M potassium phosphate buffer (pH 7.4).

## Additional file

**Additional file 1.** Comparison of anaerobic growth rescue by FRE- and P_L_lacO1-based expression. Strain Δ*ldhA* Δ*frdBC* Δ*adhE* Δ*pta* transformed with the best FRE combination (FRE_*ackA*_::*atoB-adhE2-crt-hbd*, FRE_*adhE*_::*ter*, FRE_*adhE*_::*fdh*) is indicated as the “FRE (Best producer)” in the figure legend. Strain Δ*ldhA* Δ*frdBC* Δ*adhE* Δ*pta* transformed with P_L_lacO1-based plasmids (pEL11, pIM8, pCS138) is indicated as the “P_L_lacO1” in the figure legend. The resulting anaerobic growth from an initial OD_600_ < 0.03 was compared. The accompanied fermentation level of 1-butanol is also shown. Samples were taken after 24 h of anaerobic switch. M9, M9 medium; M9 0.5% YE, M9 medium with 5 g/L of yeast extract (YE).

**Additional file 2.** Primers used in this study.

## Abbreviations

FRE: fermentation regulatory element
TB: terrific broth
M9Y: M9 medium with 0.5% yeast extract
NADH: reduced nicotinamide adenine dinucleotide
CoA: coenzyme A
OD: optical density
