# Supplementary figures and images for: Self-regulated 1-butanol production in Escherichia coli based on the endogenous fermentative control
Source: Biotechnol Biofuels. 2016 Dec 19;9:267. doi: 10.1186/s13068-016-0680-1 (PMC5168855; doi:10.1186/s13068-016-0680-1)

## Slide 1
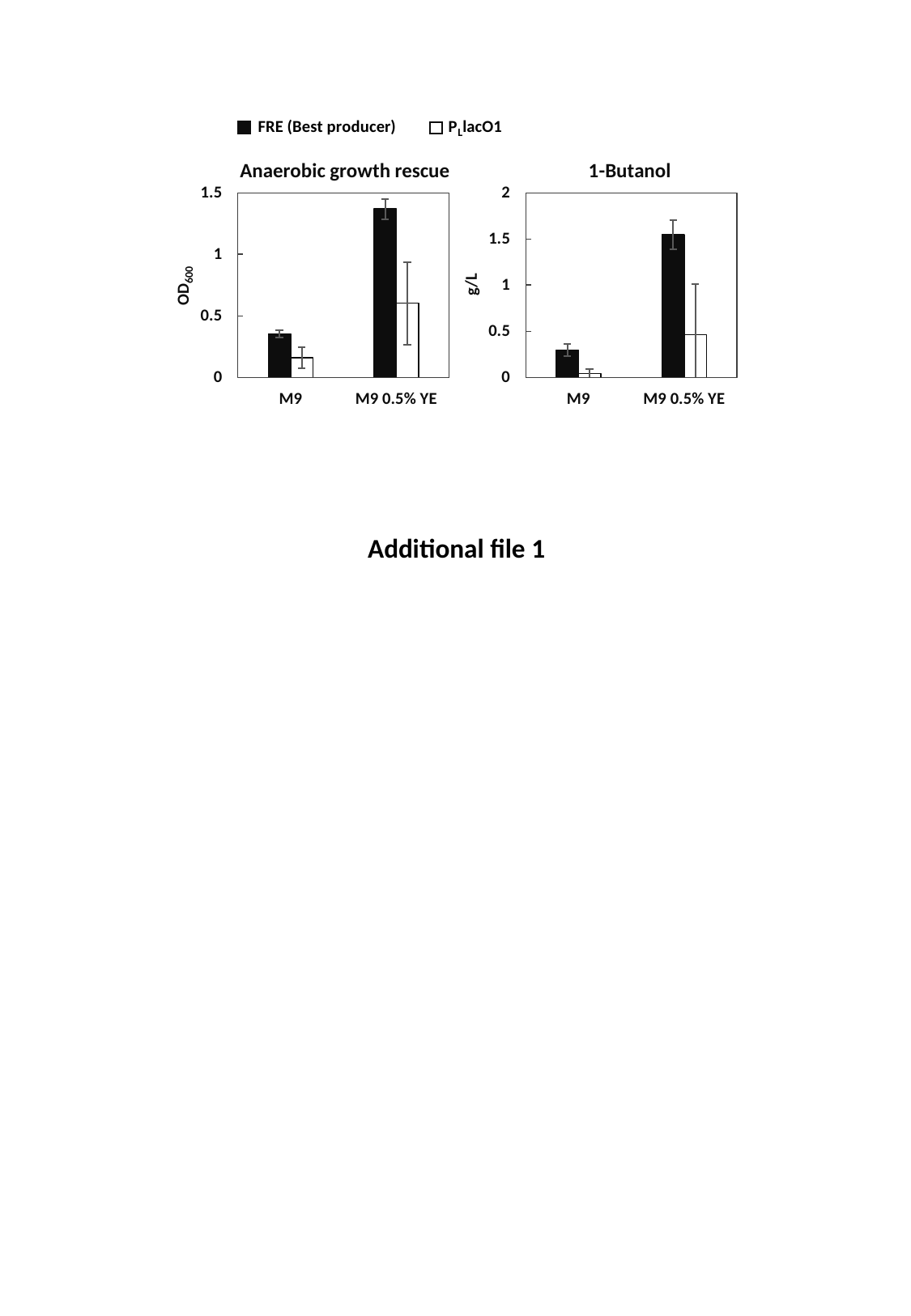

Additional file 1

Supplement: Supplementary file 1 — Additional file 1. Comparison of anaerobic growth rescue by FRE- and PLlacO1-based expression. Strain ΔldhA ΔfrdBC ΔadhE Δpta transformed with the best FRE combination (FREackA::atoB-adhE2-crt-hbd, FREadhE::ter, FREadhE::fdh) is indicated as the “FRE (Best producer)” in the figure legend. Strain ΔldhA ΔfrdBC ΔadhE Δpta transformed with PLlacO1-based plasmids (pEL11, pIM8, pCS138) is indicated as the “PLlacO1” in the figure legend. The resulting anaerobic growth from an initial OD600 < 0.03 was compared. The accompanied fermentation level of 1-butanol is also shown. Samples were taken after 24 h of anaerobic switch. M9, M9 medium; M9 0.5% YE, M9 medium with 5 g/L of yeast extract (YE). [file 13068_2016_680_MOESM1_ESM.pptx]
