# Supplementary material for: Self-regulated 1-butanol production in Escherichia coli based on the endogenous fermentative control
Source: Biotechnol Biofuels. 2016 Dec 19;9:267. doi: 10.1186/s13068-016-0680-1 (PMC5168855; doi:10.1186/s13068-016-0680-1)
Supplement: Supplementary file 2 — Additional file 2. Primers used in this study. [file 13068_2016_680_MOESM2_ESM.docx]

Additional File 2. Primers used in this study

| **Primer** | **Sequence 5’ -> 3’** |
| --- | --- |
| BuOH-1 | TAAAAATAGGCGTATCACGAGGCCCTTTCGTCTTCACTGTGCAAATTCACAACTCAGCG |
| BuOH-2 | ACGTACCGCACTGACGATGACACAATTTTTCATGGAAGTACCTATAATTGATACGTGGC |
| BuOH-3 | AAATAGGCGTATCACGAGGCCCTTTCGTCTTCACAAAGCCGGATAATGTTAGCCATAAA |
| BuOH-4 | ACGTACCGCACTGACGATGACACAATTTTTCATAATGCTCTCCTGATAATGTTAAACTT |
| BuOH-5 | AAATAGGCGTATCACGAGGCCCTTTCGTCTTCACAAATTAAGCATTCAATACGGGTATT |
| BuOH-6 | TACGTACCGCACTGACGATGACACAATTTTTCATAAGACTTTCTCCAGTGATGTTGAAT |
| BuOH-7 | ATAAAAATAGGCGTATCACGAGGCCCTTTCGTCTTCACATCAAACAGCGGTGGGCAGTG |
| BuOH-8 | TACGTACCGCACTGACGATGACACAATTTTTCATGACATTCCTCCAGATTGTTTTTATC |
| BuOH-9 | ATGAAAAATTGTGTCATCGTCAGTG |
| BuOH-10 | CTGAGCCTTTCGTTTTATTTGATGCCTCTAGATTATTTTGAATAATCGTAGAAACCTTT |
| BuOH-11 | TCAGGAAAAGGTTTCTACGATTATTCAAAATAATCTAGAGGCATCAAATAAAACGAAAG |
| BuOH-12 | GTGAAGACGAAAGGGCCTCGT |
| BuOH-13 | TAAAAATAGGCGTATCACGAGGCCCTTTCGTCTTCACTGTGCAAATTCACAACTCAGCG |
| BuOH-14 | TCCGGCATCATATAACACCAGCACAATTTTCATGGAAGTACCTATAATTGATACGTGGC |
| BuOH-15 | AAATAGGCGTATCACGAGGCCCTTTCGTCTTCACAAAGCCGGATAATGTTAGCCATAAA |
| BuOH-16 | TCCGGCATCATATAACACCAGCACAATTTTCATAATGCTCTCCTGATAATGTTAAACTT |
| BuOH-17 | ATAAAAATAGGCGTATCACGAGGCCCTTTCGTCTTCACATCAAACAGCGGTGGGCAGTG |
| BuOH-18 | TTCCGGCATCATATAACACCAGCACAATTTTCATGACATTCCTCCAGATTGTTTTTATC |
| BuOH-19 | AAATAGGCGTATCACGAGGCCCTTTCGTCTTCACAAATTAAGCATTCAATACGGGTATT |
| BuOH-20 | TTCCGGCATCATATAACACCAGCACAATTTTCATAAGACTTTCTCCAGTGATGTTGAAT |
| BuOH-21 | ATGAAAATTGTGCTGGTGTTATATG |
| BuOH-22 | GTGAAGACGAAAGGGCCTCGTG |
| BuOH-23 | TAAAAATAGGCGTATCACGAGGCCCTTTCGTCTTCACTGTGCAAATTCACAACTCAGCG |
| BuOH-24 | AATATTGTTCCTAACCATTGGTTTTACAATCATGGAAGTACCTATAATTGATACGTGGC |
| BuOH-25 | AAATAGGCGTATCACGAGGCCCTTTCGTCTTCACAAAGCCGGATAATGTTAGCCATAAA |
| BuOH-26 | AATATTGTTCCTAACCATTGGTTTTACAATCATAATGCTCTCCTGATAATGTTAAACTT |
| BuOH-27 | ATAAAAATAGGCGTATCACGAGGCCCTTTCGTCTTCACATCAAACAGCGGTGGGCAGTG |
| BuOH-28 | AAATATTGTTCCTAACCATTGGTTTTACAATCATGACATTCCTCCAGATTGTTTTTATC |
| BuOH-29 | AAATAGGCGTATCACGAGGCCCTTTCGTCTTCACAAATTAAGCATTCAATACGGGTATT |
| BuOH-30 | AAATATTGTTCCTAACCATTGGTTTTACAATCATAAGACTTTCTCCAGTGATGTTGAAT |
| BuOH-31 | ATGATTGTAAAACCAATGGTTAGGAA |
| BuOH-32 | GTGAAGACGAAAGGGCCTCGTG |
